# Subcellular Trafficking and Functional Relationship of the HSV-1 Glycoproteins N and M

**DOI:** 10.3390/v8030083

**Published:** 2016-03-17

**Authors:** Hannah Striebinger, Christina Funk, Verena Raschbichler, Susanne M. Bailer

**Affiliations:** 1 Max von Pettenkofer-Institute, Ludwig-Maximilians-University München, Munich 80336, Germany; h.striebinger@yahoo.de (H.S.); verenarasch@gmail.com (V.R.); 2 Institute for Interfacial Engineering and Plasma Technology IGVP, University of Stuttgart, Stuttgart 70569, Germany; Christina.Funk@igb.fraunhofer.de; 3 Fraunhofer Institute for Interfacial Engineering and Biotechnology IGB, Stuttgart 70569, Germany

**Keywords:** herpesvirus, HSV-1, glycoprotein N, UL49.5, glycoprotein M, UL10

## Abstract

The herpes simplex virus type 1 (HSV-1) glycoprotein N (gN/UL49.5) is a type I transmembrane protein conserved throughout the herpesvirus family. gN is a resident of the endoplasmic reticulum that in the presence of gM is translocated to the *trans* Golgi network. gM and gN are covalently linked by a single disulphide bond formed between cysteine 46 of gN and cysteine 59 of gM. Exit of gN from the endoplasmic reticulum requires the N-terminal core of gM composed of eight transmembrane domains but is independent of the C-terminal extension of gM. Co-transport of gN and gM to the *trans* Golgi network also occurs upon replacement of conserved cysteines in gM and gN, suggesting that their physical interaction is mediated by covalent and non-covalent forces. Deletion of gN/UL49.5 using bacterial artificial chromosome (BAC) mutagenesis generated mutant viruses with wild-type growth behaviour, while full deletion of gM/UL10 resulted in an attenuated phenotype. Deletion of gN/UL49.5 in conjunction with various gM/UL10 mutants reduced average plaque sizes to the same extent as either single gM/UL10 mutant, indicating that gN is nonessential for the function performed by gM. We propose that gN functions in gM-dependent as well as gM-independent processes during which it is complemented by other viral factors.

## 1. Introduction

Herpesviruses have evolved a life cycle that strongly depends on several membrane-associated processes [1,2]. Depending on the subfamily, herpesviruses encode more than 11 different transmembrane proteins. Of those, a rather small set consisting of glycoproteins (g) gB, gH, gL, gM and gN is evolutionarily conserved, consistent with important roles in viral growth. While gB, gH, and gL are essential, the functional relevance of gM and gN varies greatly throughout the herpesviral families ([3]; and ref. therein). With few exceptions, gM of alphaherpesviruses is nonessential for viral growth in cultured cells [4,5,6,7,8,9,10,11,12,13]. In HSV-1, deletion of gM/UL10 attenuates viral growth [4,5,6,11,12,14]. In contrast, surprisingly little is known about the functional relevance of HSV-1 gN either alone or in conjunction with gM.

**Figure 1:**
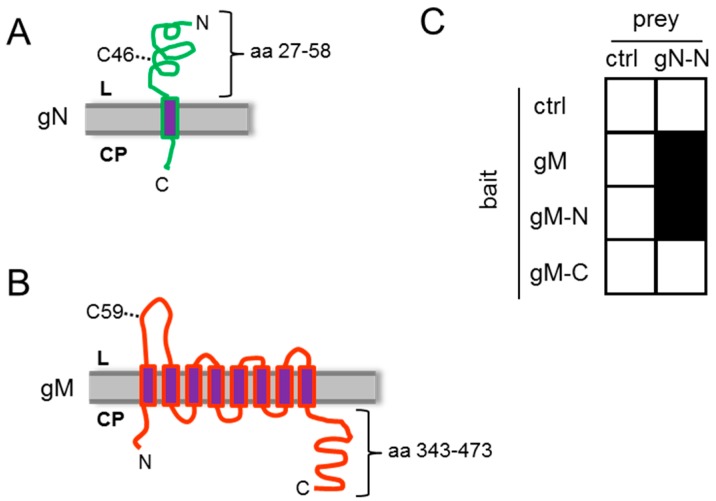
HSV-1 gN and gM interact via their N-terminal domains. (**A**) The schematic figure shows the putative localization of HSV-1 gN within cellular membranes. The N-terminal domain faces the luminal (L) side and the C-terminal domain is exposed on the cytoplasmic (CP) side, with a membrane anchor domain near the C terminus (CBS SignalP and TMHMM Server were used for prediction); (**B**) HSV-1 gM is a membrane protein encoding 8 predicted membrane domains with both N- and C-terminal domains exposed to the cytoplasm; (**C**) the interacting domains of gM with gN were analyzed by applying the yeast-2-hybrid system. Yeast cells expressing either full-length gM or gM 1–342 (gM–N) fused to Gal4–DBD together with Gal4–AD-coupled N-terminal domain of gN 27–58 (gN–N) were able to grow on medium selective for reporter gene activity, while the cytoplasmic tail of gM 343–473 (gM–C) failed to interact. As a control (ctrl) empty bait and prey plasmids were used.

HSV-1 gN is encoded by the ORF UL49.5 and comprised of 91 amino acids (aa). It is a type I transmembrane protein with a single transmembrane-spanning domain. The preferred model predicts a topology where the larger N-terminal domain is exposed to the lumen of the endoplasmic reticulum (ER)/*trans* Golgi network (TGN), while the C-terminal end is oriented towards the cytosol (Figure 1A). HSV-1 gM, a type III transmembrane protein encoded by the ORF UL10, is predicted to contain eight membrane-spanning domains with both the N- and C-terminal ends oriented towards the cytosol (Figure 1B; [14,15]). In the course of infection, HSV-1 gM is targeted to the nuclear envelope, to perinuclear virions, the TGN, and extracellular virions [15,16,17,18,19]. Its ability to modulate membrane fusion induced by the viral proteins gB, gH/gL, and gD hints at a role of gM in entry [15,20,21,22,23]. Several lines of evidence support a complex formation of gN with gM ([24]; and ref. therein). A disulphide bridge was reported to be formed between several gM and gN orthologs [3,25,26,27]. Furthermore, gM seems to partner with gN to modulate the fusion machinery ([24], and ref. therein).

We aimed to decipher the physical interaction of HSV-1 gN and gM, the subcellular localization of gN in association with gM variants and the functional relationship of gN with gM. We found that gN is an ER resident and that exit of gN from the endoplasmic reticulum requires the N-terminal core of gM composed of eight transmembrane domains but is independent of the C-terminal extension of gM. gN and gM physically interact via their N-terminal domains, and this interaction involves covalent and non-covalent forces. BAC mutagenesis revealed that gN is nonessential and genetically independent of gM. Based on our data, we propose that, while gN and gM physically interact, their interaction seems to be nonessential and potentially compensated by other factors.

## 2. Materials and Methods

### 2.1. Cells and Viruses

HeLa (ATCC CCL-2™) and Vero cells (ATCC CRL-1587™) were cultured in Dulbecco’s modified Eagle medium (DMEM) containing 10% fetal calf serum (FCS). The strain HSV1(17^+^)lox (kindly provided by Beate Sodeik) was used for all experiments and as a PCR template. Plasmid and BAC transfections were performed using Effectene (Qiagen, Hilden, Germany) and Lipofectamine 2000 (Invitrogen, Darmstadt, Germany), respectively.

### 2.2. Yeast 2-Hybrid System

The bait plasmid pGBKT7 and the prey plasmid pGADT7 were transformed into the yeast strains Y187 and AH109, respectively (BD Biosciences/Clontech, Palo-Alto, CA, USA). Diploid cells generated by mating of the haploid strains were selected for both plasmids, and interaction of the encoded proteins was detected by growth of diploid cells on selective media essentially as described in the manufacturer’s protocol (Clontech).

### 2.3. Plasmids

The full-length (FL) ORF of UL49.5, UL10 and the respective fragments were amplified by nested PCR using primers listed in Table 1. Gateway recombinational cloning was performed as described in the manufacturer’s protocol (Invitrogen), and the primers used were equipped with attB sites (Table 1, underlined). PCR products were cloned into the entry vector pDONR207 and subsequently transferred into pGBKT7, pGADT7, pCR3–N–HA or p–C–EYFP destination vectors. Single base pair exchanges (bold) were introduced into HA-tagged constructs using the QuikChange Site-directed Mutagenesis Kit (Agilent Technologies, Waldbronn, Germany). Plasmids encoding the cysteine mutants HA–gM–C59A and gN–C46A–EYFP were generated likewise.

### 2.4. BAC Mutagenesis

BAC mutagenesis was carried out essentially as described elsewhere [14,28]. Briefly, mutants of UL10/gM were generated by disrupting three methionine codons that serve as potential start codons for protein synthesis at the beginning of the open reading frame (ORF) UL10. While Lox-UL10_mt1/gM 19–473 carries a premature stop codon at position 3 of ORF UL10, Lox-UL10_mt2/gM 133/135–473 has an additional point mutation leading to expression of isoleucine instead of methionine at position 19 (M19I). Finally, insertion of the *galK*-kan-cassette into Lox-UL10_mt3/ΔgM leads to full disruption of the ORF UL10 at position 150. Deletion of UL49.5/gN was obtained by introducing stop codons at positions 5 and 6. For double mutants, the mutation of UL49.5/gN was combined with UL10/gM mt1, 2 or 3.

### 2.5. Analysis of Virus Growth

HSV-1 propagation, titration and kinetics were done as described [28]. To determine plaque sizes, a monolayer of Vero cells was incubated (1 h, 37 °C, 5% CO_2_) in triplicates with virus dilution, resulting in a multiplicity of infection (MOI) of 0.1, and then overlaid with medium containing 0.75% carboxymethylcellulose and incubated for 3 days. Subsequently, cells were fixed and stained with 0.2% crystal violet solution (10 min). The resulting plaques were imaged using light microscopy at 100-fold magnification, and the plaque area of 9 plaques was subsequently determined in square pixel with Adobe Photoshop software.

### 2.6. Cell Lysis, Reducing and Non-Reducing Gel Electrophoreses and Western Blotting

HeLa cells were grown in 6 wells and transfected with single plasmids encoding gN–EYFP, HA–gM, gN–C46A–EYFP, HA–gM–C59A or combinations of them using Effectene according to the manufacturer’s protocol (Qiagen). 24 h post-transfection, the cells were incubated for 10 min with a RIPA-buffer (0.1 M Tris-HCL pH 7.4; 0.75 M NaCl; 5% (*w/v*) Triton X-100; 5% (*w/v*) Sodium deoxycholate; 5% SDS; 1x complete Protease-Inhibitor Cocktail (Roche Diagnostics, Mannheim, Germany)) on ice, collected using a cell scraper and again incubated for 10 min on ice. The lysates were cleared by centrifugation (13,000 rpm, 10 min, 4 °C) and analyzed by SDS-PAGE under reducing (with Dithiotreitol (DTT)), as well as non-reducing (without DTT) conditions, followed by Western blotting with anti-HA and anti-GFP antibodies together with peroxidase-conjugated secondary antibodies.

### 2.7. Indirect Immunofluorescence Analysis and Microscopy

Transfected HeLa cells on coverslips were fixed with 2% formaldehyde/PBS (15 min) 20 h post-transfection (h.p.t.) and permeabilized with 0.5% Triton X-100 (5 min, 4 °C). Mouse monoclonal antibodies anti-HA (Santa Cruz Biotechnology, Heidelberg, Germany) and rabbit polyclonal antibodies anti-TGN46 (AbD Serotec) as well as anti-calreticulin (Sigma-Aldrich, Steinheim, Germany) were used together with fluorescently labeled secondary antibody (Invitrogen). gN–EYFP was visualized directly. Cells were examined using a Leica or Zeiss confocal microscope, and pictures were processed using Adobe Photoshop or Zen Lite.

## 3. Results

### 3.1. HSV-1 gN and gM Interact via Their N-Terminal Domains

The HSV-1 glycoprotein gN is a type I transmembrane protein with a single transmembrane-spanning domain (Figure 1A). A short N-terminal signal peptide is believed to mediate its co-translational insertion into the endoplasmic reticulum (ER), resulting in the N-terminal domain exposed to the ER lumen. HSV-1 gM is a type III membrane protein containing 8 predicted transmembrane-spanning domains with both the N- and C-terminal ends oriented towards the cytosol (Figure 1B). To analyze the potential interaction of gN and gM, the yeast-2-hybrid (Y2H) assay was applied. To this end, the N-terminal domain of gN 27–58 (gN–N) lacking the predicted signal peptide (aa 1–26) as well as the transmembrane anchor (aa 59–91) was expressed fused to the Gal4–AD (activation domain; prey). As bait, the full-length gM (gM 1–473), the N-terminal domain of gM 1–342 (gM–N) or the cytoplasmically exposed C-terminal domain gM 343–473 (gM–C) was expressed as fusion with the Gal4-DBD (DNA-binding domain; bait). Yeast cells expressing gN–N together with full-length gM or the N-terminal domain gM–N were able to grow on media selective for reporter gene activity (Figure 1C). We thus conclude that gN and gM physically interact and that this interaction is mediated by their N-terminal domains.

### 3.2. HSV-1 gN is an ER Resident that Requires the Hydrophobic Core of gM for Transport to the TGN

**Figure 2:**
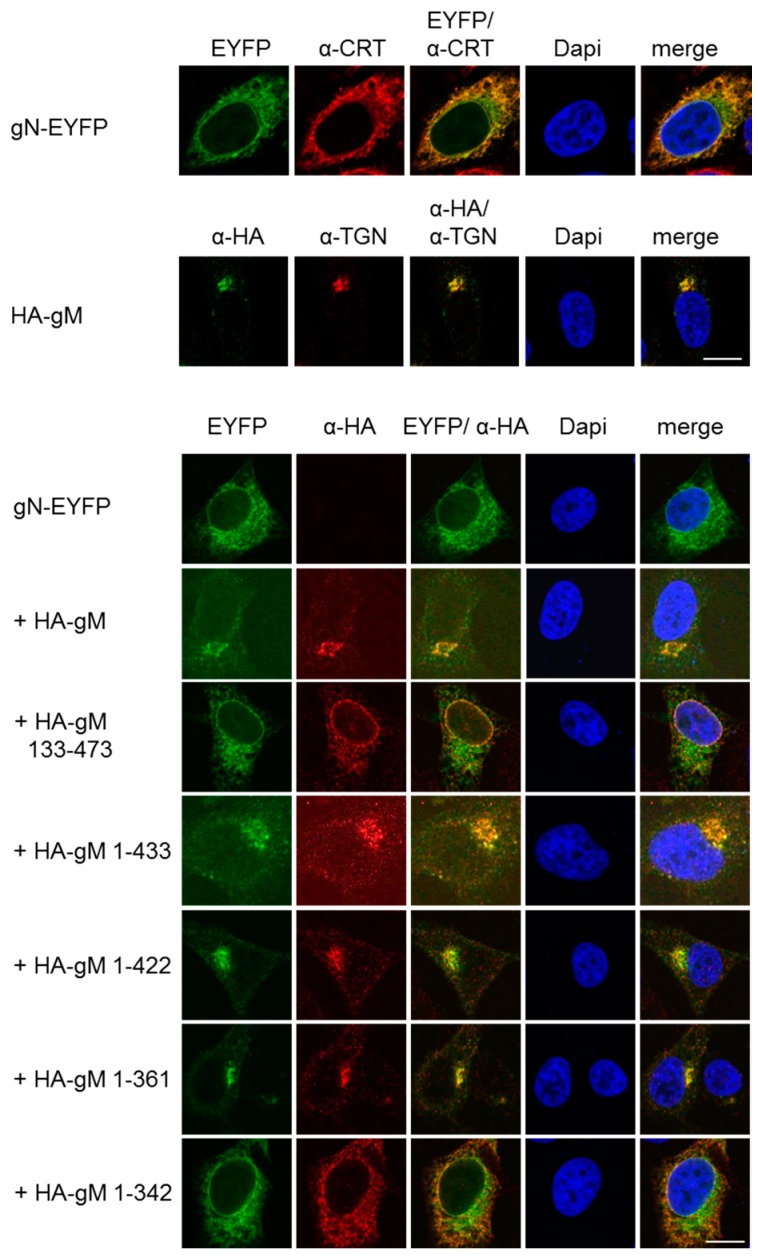
HSV-1 gN is an ER resident that requires the hydrophobic core of gM for transport to the TGN. To follow their subcellular distribution, gN–EYFP or HA–gM were transiently expressed either alone or combining gN–EYFP with either full-length (HA–gM) or N- and C-terminal truncation mutants of gM (HA–gM 133–473, HA–gM 1–433, HA–gM 1–422, HA–gM 1–361, HA–gM 1–342) in HeLa cells for 20 h. Anti-HA antibodies were used to detect the localization of gM and gM truncations, followed by secondary reagents. gN–EYFP was visualized directly. Antibodies recognizing the marker proteins Calreticulin (CRT) and TGN46 were used to visualize the ER and the TGN, respectively. Nuclei were visualized by DAPI staining. The scale bars correspond to 10 µm.

To analyze the subcellular localization of gN, a plasmid encoding gN with the C-terminal end fused to the enhanced yellow fluorescent protein (gN–EYFP) was transiently transfected into HeLa cells and analyzed 20 h.p.t. gN–EYFP was exclusively located to the cytoplasm and showed a reticular pattern of distribution, revealing that gN is an ER resident when expressed in the absence of other viral factors (Figure 2). When co-expressed with gM, a resident of the *trans* Golgi network (TGN; [14]), both proteins co-localized in a perinuclear area reminiscent of the TGN. Thus, consistent with previous reports [24], in the presence of gM, gN is able to leave the ER and reach the TGN.

We recently generated several gM truncation mutants and showed that a core gM consisting of all eight transmembrane domains is able to leave the ER and to reach the TGN [14]. To determine the minimal region of gM required for transport of gN to the TGN, gN–EYFP was transiently expressed in HeLa cells together with various gM truncation mutants and analyzed 20 h.p.t. If co-expressed with C-terminal truncation mutants of gM (gM 1–433, gM 1–422, or gM 1–361), gN–EYFP was targeted to the TGN along with either of the gM variants. In contrast, if co-expressed with the mutant gM 133–473 that lacks the first two hydrophobic domains or the mutant gM 1–342 where the last transmembrane domain is compromised due to the absence of the last amino acid that acts as membrane transfer stop signal, gN–EYFP was unable to exit the ER (Figure 2). These results showed that, consistent with the Y2H data (Figure 1C), gN and gM interact via their N-terminal domains and that an intact hydrophobic core of gM is required and sufficient to translocate gN to the TGN.

### 3.3. A Non-Covalent Interaction of HSV-1 gN with gM is Sufficient for its Transport to the TGN

**Figure 3:**
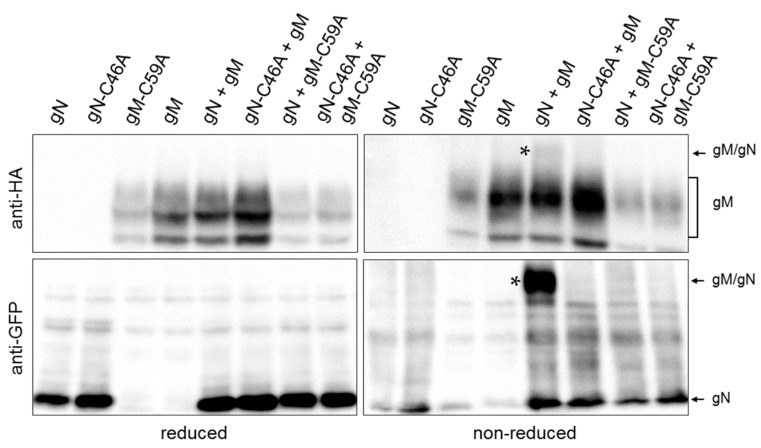
HSV-1 gN forms a covalent interaction with gM by disulphide bonding between C59 of gM and C46 of gN. To determine whether C59 of gM and C46 of gN form a disulphide bridge, site-directed mutagenesis was performed to replace cysteines (C) by alanines (A). Plasmids encoding gN–EYFP, HA–gM, HA–gM–C59A and gN–C46A–EYFP were transiently transfected into HeLa cells either alone or in combination. Cell extracts were analyzed 24 h.p.t. using reducing and non-reducing SDS-PAGE followed by Western blotting. Proteins were detected using anti-HA and anti-GFP antibodies followed by peroxidase-conjugated secondary antibodies. The complex formed between gN and gM is marked by stars.

Interaction of gN with gM supported by an N-terminal disulphide bridge has been reported for several orthologs [3,25,26,27]. Likely candidates for disulphide bonding are cysteine 46 (C46) within the ectodomain of gN (Figure 1A) and cysteine 59 (C59) of gM exposed to the ER lumen via a loop between the first and second hydrophobic domain (Figure 1B). To determine whether HSV-1 gN and gM are covalently linked by a disulphide bond, HeLa cells were transiently transfected with plasmids encoding HA–gM and/or gN–EYFP and analyzed 20 h.p.t. Biochemical analysis using reducing and non-reducing SDS-PAGE followed by Western blotting revealed that under non-reducing but not under reducing conditions, co-expressed gN–EYFP and HA–gM are delayed in gel migration, indicative of complex formation by disulphide bonding (Figure 3). To identify the cysteines involved in disulphide bonding, C59 of gM and C46 of gN were replaced by alanines (A) using site-directed mutagenesis. Plasmids encoding HA–gM, HA–gM–C59A, gN–EYFP, and gN–C46A–EYFP were transiently transfected either alone or in combinations (Figure 3). Unlike upon co-expression of gN and gM, neither co-expression of gM and gN–C46A–EYFP nor gN–EYFP and HA–gM–C59A resulted in proteins delayed in migration. Together these results show that C59 of gM and C46 of gN are required and sufficient for disulphide bonding of HSV-1 gN and gM.

**Figure 4:**
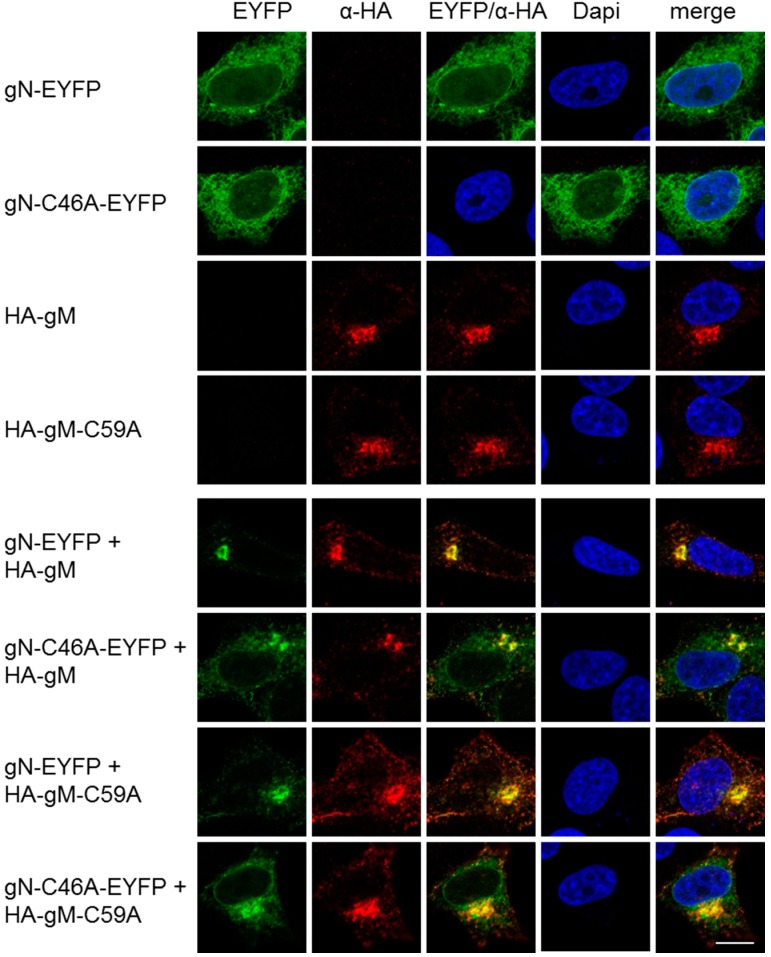
A non-covalent interaction of HSV-1 gN with gM is sufficient for its transport to the TGN. The subcellular localizations of gN–EYFP, gN–C46A–EYFP, HA–gM or HA–gM–C59A were analyzed alone, alternatively, combinations of either wild type with the corresponding mutant or of both mutants were analyzed by transient expression in HeLa cells for 20 h. Anti-HA antibodies were used to detect the localization of HA–gM and HA–gM–C59A followed by secondary reagents. gN–EYFP and gN–C46A–EYFP were visualized directly. Nuclei were visualized by DAPI staining. The scale bar corresponds to 10 µm.

To determine the importance of these cysteines for co-transport of gN with gM to the TGN, plasmids encoding HA–gM–C59A and gN–C46A–EYFP were transiently transfected into HeLa cells and analyzed 20 h.p.t. Fluorescence analysis revealed that gN–C46A–EYFP was located to the cytoplasm and distributed in a reticular pattern comparable to gN–EYFP (Figure 4). HA–gM–C59A was found at the TGN reminiscent of the wild-type HA–gM (Figure 4). Upon co-expression of either mutant with the corresponding wild type or of both mutants, translocation of the gN variant to the TGN was observed. We thus conclude that a non-covalent interaction of HSV-1 gN with gM is sufficient for its transport to the TGN.

### 3.4. During Infection, HSV-1 gN is Nonessential and Redundant in Association with gM

**Figure 5:**
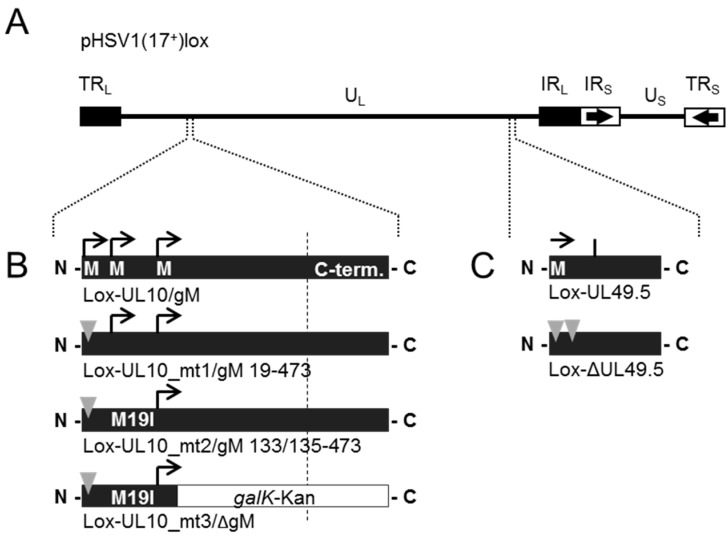

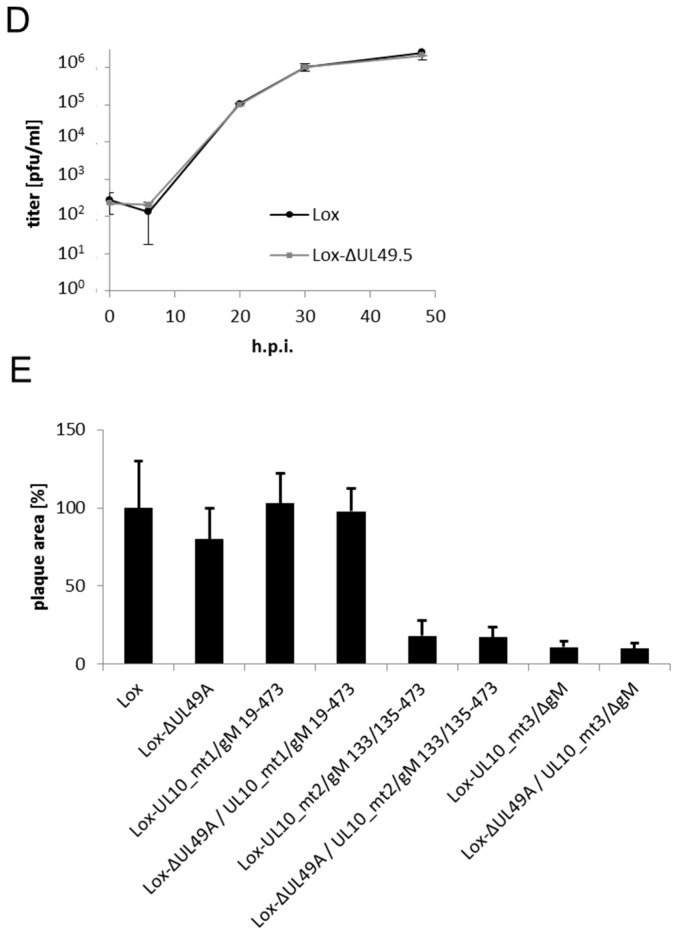
During infection, HSV-1 gN is nonessential and redundant in association with gM. (**A**) The schematic diagram of the pHSV1(17^+^)lox genome with the unique long (UL) and unique short (US) region flanked by the terminal repeat (TR) and internal repeat (IR) regions shows the spatial distribution of the glycoprotein encoding genes UL10 (glycoprotein M, gM) and UL49.5 (glycoprotein N, gN); (**B**) Generation of HSV-1 gM mutants by interfering with three methionines that might serve as potential start codons for protein synthesis at the beginning of the open reading frame (ORF) UL10 [14]. Deletion of gM in Lox-UL10_mt1/gM 19–473 was obtained by base pair exchanges that result in a premature stop codon at position 3 of ORF UL10. In Lox-UL10_mt2/gM 133/135–473, an additional mutation leads to expression of isoleucine instead of methionine at position 19 (M19I), while insertion of the *galK*-kan-cassette into Lox-UL10_mt2/gM 133/135–473 leads to disruption of ORF UL10 at position 150; (**C**) Generation of HSV-1 gN deletion mutant Lox-ΔUL49.5 by introducing base pair exchanges resulting in two premature stop codons at positions 5 and 6. Furthermore, double deletion mutants of gN and gM were produced by combining the deletion of ΔUL49.5 with each mutation of UL10 (mt1, mt2, mt3); (**D**) To compare the growth properties of Lox-ΔUL49.5 to the parental Lox virus, Vero cells were infected at an MOI of 0.1, the supernatant was harvested at the indicated time points and titrated on Vero cells in triplicates; (**E**) The plaque morphology of each gM or gN single or double mutant was analyzed in comparison to wild-type virus (Lox). The plaque area produced by infection of Vero cells with the different virus strains was analyzed microscopically 3 days post-infection. Plaque areas were determined, mean value and standard deviation were calculated.

To determine the functional importance of HSV-1 ORF UL49.5/gN for viral growth, a deletion mutant Lox-ΔUL49.5 was generated using BAC mutagenesis as previously described ([28]; Figure 5A,C). The BAC Lox-ΔUL49.5 could readily be reconstituted upon transfection of Vero cells. Thus, UL49.5/gN is nonessential for viral growth *in vitro*. Since the glycoproteins gN and gM interact, the effect of simultaneous mutagenesis of both genes was analyzed likewise. The mutational strategy of UL10/gM was as reported ([14,28]; Figure 5A,B) and is based on preventing protein synthesis initiated at codon 1 (Lox-UL10_mt1/gM 19–473) or an alternative start codon at position 19 (Lox-UL10_mt2/gM 133/135–473), while insertion of the *galK*-kan-cassette into Lox-UL10_mt2/gM 133/135–473 leads to disruption of ORF UL10 at position 150 (Lox-UL10_mt3/ΔgM). While Lox-UL10_mt1/gM 19–473 showed wild-type-like growth characteristics, Lox-UL10_mt2/gM 133/135–473 and Lox-UL10_mt3/ΔgM, although viable, were compromised in growth [14]. The double mutants Lox-ΔUL49.5/UL10_mt1/gM 19–473, Lox-ΔUL49.5/UL10_mt2/gM 133/135–473 and Lox-ΔUL49.5/UL10_mt3/ΔgM reflected the growth characteristics of the single mutants Lox-UL10_mt1/gM 19–473, Lox-UL10_mt2/gM 133/135–473 and Lox-UL10_mt3/ΔgM, respectively.

For detailed analysis of the growth properties of our novel UL49.5 deletion mutant, Vero cells were infected in triplicates at a multiplicity of infection (MOI) of 0.1 with progeny virus of Lox-ΔUL49.5 and the parental Lox (Figure 5D). Supernatants harvested at the indicated time points were titrated on Vero cells. The mutant Lox-ΔUL49.5 and the parental Lox showed similar growth behaviour, suggesting that entry and subsequent steps of viral replication were unaffected by deletion of the UL49.5 gene. Consistently, plaques formed by the Lox-ΔUL49.5 virus were comparable to the parental strain (Figure 5E). Together our data suggest that gN is nonessential for the function performed by gM. Potentially, gN functions in gM-dependent as well as gM-independent processes during which it is complemented by viral proteins yet to be identified.

## 4. Discussion

Despite being among the five evolutionarily conserved HSV-1 glycoproteins, surprisingly little is known about the subcellular localization and functional relevance of HSV-1 gN together with gM. Consistent with recent data [24], we show that gN is an ER resident protein unless other viral proteins are available. In the presence of gM, gN is translocated to the TGN ([24]; this study). Furthermore, we show that translocation of gN to the TGN requires the hydrophobic core of gM [14] but is independent of the cytoplasmic C-terminal extension of gM.

Our data indicate a physical interaction between gN and the core of gM. Consistent with the interaction between orthologs [3,25,26,27], we show here that HSV-1 gN and gM are linked by a single disulphide bond formed between cysteine C46 present in the luminal domain of gN and cysteine C59 present in the largest loop of gM. Surprisingly, however, we found that disulphide bonding is not required for their co-transport to the TGN. It is thus conceivable that additional interactions exist between gM and gN. In fact, gM 134–473 that lacked the first two transmembrane domains containing the conserved cysteine still formed a complex with gN [24]. We found that all gM transmembrane domains are required for functional complementation [14], suggesting that gM forms a dense helical bundle within the endoplasmic reticulum (ER) membrane. Thus, interaction between gM and gN could involve several ER exposed loops of gM potentially together forming a platform for interaction with gN. In addition, intramembrane interactions may be established between the transmembrane segments of gN and gM. Taken together, we conclude that the gM/gN complex can form by covalent as well as non-covalent associations reminiscent of reports on the HCMV orthologous pair [3].

In the course of infection, HSV-1 gM is targeted to the nuclear envelope, to perinuclear virions, the TGN, as well as mature virions [15,16,17,18,19]. Due to the lack of specific antibodies to gN, we can merely speculate about the localization of gN during infection. Potentially, various subpopulations of gN and gM may co-exist, gM–gN in complex with each other, as well as gN and gM proteins that travel independently of each other, thereby performing specific functions depending on the compartments where they reside. Surprisingly, however, HSV-1 gN could not be detected in virions using mass spectrometry [29]. Considering that gM and gN form a complex [24], it is likely that gN escaped detection due to its small size which is unsuited to generate appropriate tryptic peptides. This assumption is further supported by the presence of gN of Pseudorabies Virus, another alphaherpesvirus, in extracellular virions [30].

Physical interaction of gM and gN suggests their functional cooperation (this study; [24]). We found that HSV-1 gN is nonessential for viral growth in a wild-type background. Furthermore, the double mutants Lox-ΔUL49.5/UL10_mt1/gM 19–473, Lox-ΔUL49.5/UL10_mt2/gM 133/135–473 and Lox-ΔUL49.5/UL10_mt3/ΔgM reflected the growth characteristics of each single UL10 mutant. These data together suggest that gN is nonessential for the function performed by gM. Recent transfection-infection experiments nevertheless point to a functional co-operation of gN and gM [24]. Transient expression of gN–EYFP followed by infection with HSV-1 resulted in syncytia, indicating a role of gN–EYFP in membrane fusion. Consistent with a functional interaction of gN and gM, fusogenic activity of gN–EYFP was reduced upon superinfection with the HSV-1 gM∆2, a mutant expressing an N-terminally truncated nonfunctional fragment of gM that is unable to exit the ER [14]. Since this gM fragment still interacts with gN [24], both proteins may be retained in the ER and thus be unable to reach sites of membrane fusion (this study). While overexpression of gN during the transfection-infection assay may create a “forced situation,” this assay could represent a valuable system to identify the domains of gM required for gM–gN-mediated membrane fusion. Unfortunately, the viral strain HSV1(17^+^)lox used in a comparable transfection-infection experiment was unable to show a role of gN in modulating the fusion machinery (data not shown). Consistently, entry of the HSV1(17^+^)lox ΔUL49.5 virus was unimpaired and comparable to the wild-type strain (data not shown). Thus, overall the proposed gM–gN-mediated fusogenic activity remains controversial ([24], and ref. therein).

Our data could also point to a function of gN performed independently of gM but functionally redundant with unknown viral partners. Interestingly, gN orthologs of varicelloviruses were recently shown to block the maturation of the major histocompatibility complex class I (MHC-I) by inhibiting the transporter associated with antigen processing (TAP) and the peptide transport into the ER [31,32,33,34]. HSV-1 gN, if transiently expressed, was unable to block the MHC-I [35]. This finding correlates well with the lack of a phenotype of the HSV-1 UL49.5 deletion mutant and may indicate that in the viral context, gN indeed cooperates with one or several viral protein(s) for immune evasion. Thus, HSV-1 gN could be part of an immune evasion strategy making infected cells invisible to cytotoxic T cells and allowing the virus to replicate. Interestingly, an analysis of bovine herpesvirus Type I (BHV-1) revealed that TAP inhibition and interaction of gN with gM as well as incorporation of both proteins into the envelope of extracellular virions are independent properties, indicating that gN could perform two different functions, one in secondary envelopment and the other in TAP inhibition [34]. Furthermore, while gN is nonessential *in vitro*, it could play an important role *in vivo* as part of an intricate balance between host immune response and immune evasion strategies of the virus. We propose that gN performs functions in association with gM but also with other as-yet-unknown viral proteins. These data provide the molecular basis for future *in vitro* and *in vivo* studies on a role of gN in virion envelopment and viral immune evasion.

## 5. Conclusions

• The evolutionarily conserved transmembrane protein gN of HSV-1 is an ER resident.
• gM and gN are covalently linked by a single disulphide bond formed between cysteine 46 of gN and cysteine 59 of gM
• HSV-1 gN and gM interact via their N-terminal halves, an interaction sufficient for transport of gN to the TGN.
• Non-covalent interaction of HSV-1 gN with gM is sufficient for its transport to the TGN.
• HSV-1 gN/UL49.5 is nonessential *in vitro*.
• HSV-1 gN/UL49.5 likely functions in gM-dependent as well as gM-independent processes.

## Figures and Tables

**Table 1:** Primer sequences used for plasmid cloning and BAC mutagenesis.

Primer Name	Primer Sequence
attB_5′	ggggacaagtttgtacaaaaaagcaggct
attB_3′	ggggaccactttgtacaagaaagctgggt
UL49.5–FL_5′	aaaaagcaggctccgccatgggcccccccagaag
UL49.5–FL_3′	agaaagctgggtctaggcgtgcccggcagc
UL49.5–Y2H_3′	
UL49.5–Y2H_5′	aaaaagcaggctccgccatgccgcgcggggagccg
UL49.5–IF_3′	agaaagctgggtggcgtgcccggcagc
UL10–FL_5′	aaaaagcaggctccgccatgggacgcccggcccccagag
UL10–Y2H_5′	
UL10–Y2H_5′–N	
UL10–FL_3′	agaaagctgggtctaccaacggcggacggtgc
UL10–Y2H_3′	
UL10–Y2H_3′–C	
UL10–∆C_3′	agaaagctgggtctaggtgcagcggagcacggccatgc
UL10–Y2H_3′–N	
UL10–C_5′	aaaaagcaggctccgccatgcgcgcctatctgtatcac
UL10–Y2H_5′–C	
UL10–M19I_5′	cagaggatctcccgactccgcgccccccacgaaaggcataaccggggcgcggac
UL10stop362aa_5′	gcgcatgcgcgactagcgacaccgcgcac
UL10stop362aa_3′	gtgcgcggtgtcgctagtcgcgcatgcgc
UL10stop423aa_5′	ggggagccgatttaggacgaggtggcg
UL10stop423aa_3′	cgccacctcgtcctaaatcggctcccc
UL10stop434aa_5′	cgaccaaaccgacgtatagtacgccaagatacaacac
UL10stop434aa_3′	gtgttgtatcttggcgtactatacgtcggtttggtcg
gM–C59A_5′	cacggtttcccgccttttacgccacggcg
gM–C59A_3′	gtggcgtaaaaggcgggaaaccgtgcccg
gN–C46A_5′	gggcgcgcgggccgagacccaaaacactg
gN–C46A_3′	gttttgggtctcggcccgcgcgcccccgatc
H5–gM/gk	tccgcgctagcgatacgctcgacgtgtactgttcgcactcgtcgtccccacctgttgacaattaatcatcggca
H3–gM/gk	caccacggtcgggttaaacacaaacggtttattaaaacggaaccaaacaggccagtgttacaaccaattaacc
UL10–mt1_5′	gcgctagcgatacgctcgacgtgtactgttcgcactcgtcgtccccaatgggatgaccggcccccagaggatctcccg
UL10–mt1_3′	caccacggtcgggttaaacacaaacggtttattaaaacggaaccaaacagctaccaacggcggacggtgc
H5–gM–mt3/gK	gccatgccgcacgccacgctgatcgccggaaacgtctgctcttggttgctcctgttgacaattaatcatcggca
H5–UL49.5/gK	cccaacacatagcaggccgcgggcccggcgtccgcgtggagcatgcggagggcctgttgacaattaatcatcggca
H3–UL49.5/gK	aagtcctgggacaccctccacccccacccctcaccccacacagggcgggtgccagtgttacaaccaattaacc
ΔUL49.5_5′	cccaacacatagcaggccgcgggcccggcgtccgcgtggagcatgcggagggatgggccccccctagtaggtct
ΔUL49.5_3′	aagtcctgggacaccctccacccccacccctcaccccacacagggcgggttcaggcgtgcccggcagccagt
